# Investigating Health Impacts of Natural Resource Extraction Projects in Burkina Faso, Ghana, Mozambique, and Tanzania: Protocol for a Mixed Methods Study

**DOI:** 10.2196/17138

**Published:** 2020-04-08

**Authors:** Andrea Farnham, Hermínio Cossa, Dominik Dietler, Rebecca Engebretsen, Andrea Leuenberger, Isaac Lyatuu, Belinda Nimako, Hyacinthe R Zabre, Fritz Brugger, Mirko S Winkler

**Affiliations:** 1 Swiss Tropical and Public Health Institute Basel Switzerland; 2 University of Basel Basel Switzerland; 3 Manhiça Health Research Centre Maputo Mozambique; 4 Swiss Federal Institute of Technology Zurich Switzerland; 5 Ifakara Health Institute Dar es Salaam United Republic of Tanzania; 6 University of Health and Allied Sciences Ho Ghana; 7 Research Institute of Health Sciences Ouagadougou Burkina Faso

**Keywords:** health impact assessment, environmental health, mixed methods, extractive industry, DHIS2, time series, GIS, cost-benefit, DHS

## Abstract

**Background:**

Natural resource extraction projects offer both opportunities and risks for sustainable development and health in host communities. Often, however, the health of the community suffers. Health impact assessment (HIA) can mitigate the risks and promote the benefits of development but is not routinely done in the developing regions that could benefit the most.

**Objective:**

Our study aims to investigate health and health determinants in regions affected by extractive industries in Burkina Faso, Ghana, Mozambique, and Tanzania. The evidence generated in our study will inform a policy dialogue on how HIA can be promoted as a regulatory approach as part of the larger research initiative called the HIA4SD (Health impact assessment for sustainable development) project.

**Methods:**

The study is a concurrent triangulation, mixed methods, multi-stage, multi-focus project that specifically addresses the topics of governance and policy, social determinants of health, health economics, health systems, maternal and child health, morbidity and mortality, and environmental determinants, as well as the associated health outcomes in natural resource extraction project settings across four countries. To investigate each of these health topics, the project will (1) use existing population-level databases to quantify incidence of disease and other health outcomes and determinants over time using time series analysis; (2) conduct two quantitative surveys on mortality and cost of disease in producer regions; and (3) collect primary qualitative data using focus groups and key informant interviews describing community perceptions of the impacts of extraction projects on health and partnership arrangements between the projects and local and national governance. Differences in health outcomes and health determinants between districts with and without an extraction project will be analyzed using matched geographical analyses in quasi-Poisson regression models and binomial regression models. Costs to the health system and to the households from diseases found to be associated with projects in each country will be estimated retrospectively.

**Results:**

Fieldwork for the study began in February 2019 and concluded in February 2020. At the time of submission, qualitative data collection had been completed in all four study countries. In Burkina Faso, 36 focus group discussions and 74 key informant interviews were conducted in three sites. In Ghana, 34 focus group discussions and 64 key informant interviews were conducted in three sites. In Mozambique, 75 focus group discussions and 103 key informant interviews were conducted in four sites. In Tanzania, 36 focus group discussions and 84 key informant interviews were conducted in three sites. Quantitative data extraction and collection is ongoing in all four study countries. Ethical approval for the study was received in all four study countries prior to beginning the fieldwork. Data analyses are underway and results are expected to be published in 2020 and 2021.

**Conclusions:**

Disentangling the complex interactions of resource extraction projects with their host communities requires an integrative approach drawing on many methodologies under the HIA umbrella. By using complementary data sources to address the question of population health in project areas from several angles, bias and missing data will be reduced, generating high-quality evidence to aid countries in moving toward sustainable development.

**International Registered Report Identifier (IRRID):**

DERR1-10.2196/17138

## Introduction

### Background

Natural resource extraction projects (eg, minerals, metals, oil, and gas) are major drivers of the economy in many developing countries, offering opportunities for sustainable economic and social growth for the local population, with accompanying implications for human health [1,2]. The nature of large natural resource extraction projects often causes upheaval in many sectors that affect both health and health determinants, including public health, society, and ecosystems [3-6]. Projects move in and offer new opportunities for jobs, improved infrastructure, a strengthened health system, and economic mobility [7]. However, historically the development of these projects is often instead accompanied by negative social and health outcomes in the surrounding community, often termed “the resource curse” [8-11]. These negative outcomes have included environmental contamination, strain on local water and sanitation resources from in-migration, reduced health equity, accidents and injuries caused by increased traffic, and increases in sexually transmitted, vector-borne, and chronic diseases [4-6,8,10,12-16]. The complex nature of resource extraction projects means that, in this upheaval, an integrated approach to identify potential opportunities and risks for the health of the population is needed.

Health impact assessment (HIA) is one approach that has the potential to harness the economic and social potential of extractive industries for the benefit of the local population. HIA is a method of impact assessment that uses a combination of quantitative and qualitative methods to prospectively identify the potential positive and negative health impacts of projects, policies, or programs [17]. In addition to investigating direct impacts on health, such as changes in disease and accident incidence, HIA gathers evidence on indirect impacts on the social, environmental, and institutional determinants of health, such as access to water and sanitation, food availability, and health system capacity, generating a strong evidence base from which decisions about public health policy and management can be made [18]. Despite the fact that environmental impact assessment has become accepted practice for mitigating environmental risks during project implementation [19,20], the use of HIA lags far behind [21]. Large gaps thus remain in our understanding of the complex picture of health and health equity in regions impacted by resource extraction projects. This need is particularly pressing in regions such as sub-Saharan Africa that already have low scores on the health-related targets of the Sustainable Development Goals (SDGs) [22]. This represents a wasted opportunity to turn the massive investments of the extractive industry into a net positive for the local population by minimizing negative health impacts of project implementation and maximizing the effects of the positive inflow of new resources on local and national development.

### Study Objectives

Our project aims to use the HIA toolbox across natural resource extraction project settings in Burkina Faso, Ghana, Mozambique, and Tanzania, countries acutely affected by the development of extractive industries. By doing so, the study will generate new evidence about health in regions affected by resource extraction and translate these findings into actionable policy recommendations. In addition, by partnering with local institutions and training local PhD students in HIA methodology, the project will build long-term capacity for carrying out HIA in the low- and middle-income countries where it is most needed.

Specifically, the study aims to (1) evaluate the effects of natural resource extraction projects on health-related targets and associated indicators of the SDGs that can be observed at the national and local level; (2) assess how projects interact with, and have effects on, local health systems; (3) determine the costs and benefits of projects for local and national health systems; and (4) characterize the influence projects exert on health equity in affected populations. The evidence generated in the study will inform a policy dialogue on whether and how HIA can be promoted as a regulatory approach as part of the larger research initiative called the HIA4SD (Health impact assessment for sustainable development) project [23].

## Methods

### Summary

**Figure 1:**
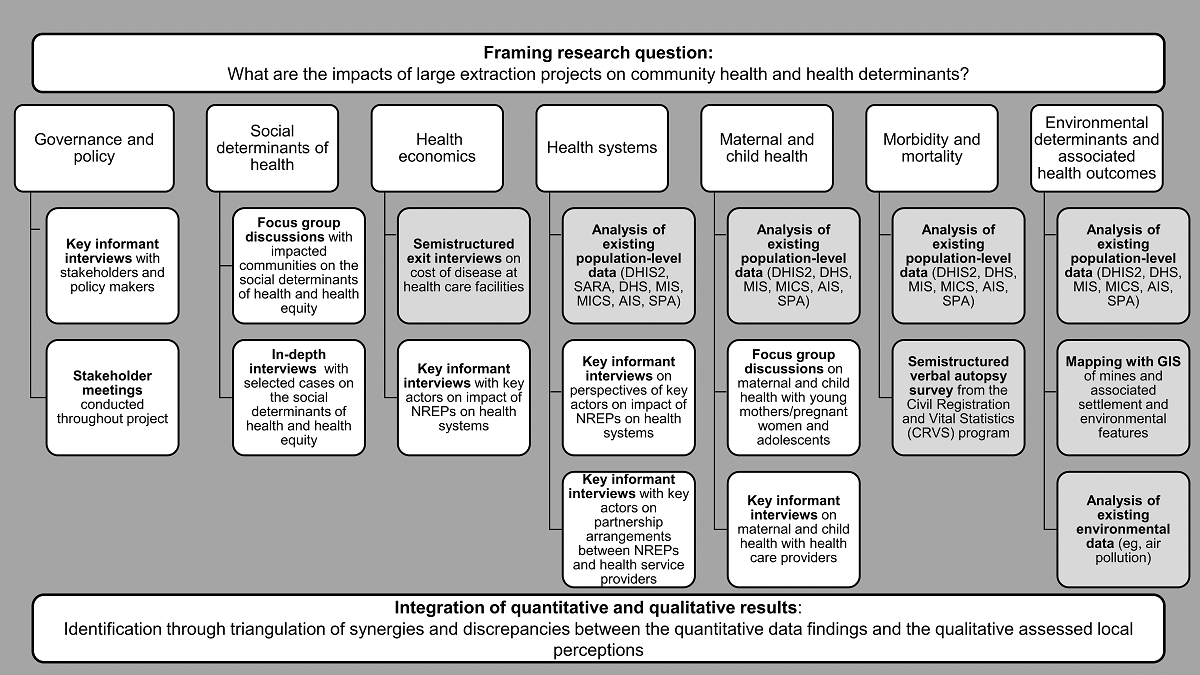
Study design. The headings are the research topics relevant to population health in natural resource extraction project (NREP) areas identified in conjunction with local partners. The specific research topics are investigated with both qualitative (white) and quantitative (gray) research methods. AIS: AIDS Indicator Survey; DHIS2: District Health Information System 2; DHS: Demographic and Health Survey; GIS: geographic information system; MICS: Multi-Indicator Cluster Survey; MIS: Malaria Indicator Survey; SARA: Service Availability and Readiness Assessment; SPA: Service Provision Assessment.

The study is designed as a concurrent triangulation mixed methods design [24], with simultaneous collection of (1) quantitative data used to measure resource extraction project effects on population health by describing incidence of disease and other health determinants and outcomes over time, as well as (2) qualitative data describing community perceptions of the impacts of projects on health. In this design, the data will be analyzed first separately and then integrated, with the advantage that each data source can complement and strengthen the findings of the other data sources, as well as drive further research questions. Based on the initial study aims, seven major research topics of interest were identified: governance and policy, social determinants of health, health economics, health systems, maternal and child health, morbidity and mortality, and environmental determinants and associated health outcomes (see Figure 1). Data will be collected on every research topic by the local team within each country and then shared across all project teams, with the aim of making comparisons both within and between each country.

### Setting

**Figure 2:**
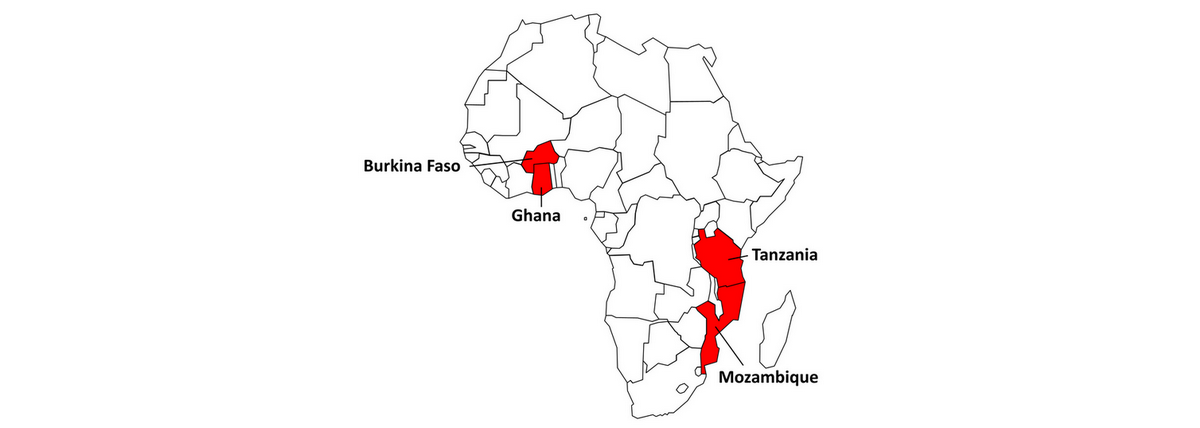
Location of study countries in sub-Saharan Africa.

The health impacts of resource extraction projects vary based on the baseline characteristics and environment of the host community [25]. Therefore, we partnered with four different countries with a history of natural resource extraction and low health-related SDG index values: Burkina Faso, Ghana, Mozambique, and Tanzania (see Figure 2). Within those countries, three to four large mining sites were chosen for qualitative and quantitative field work. The mines were chosen based on type, size, length of operation, and type of ownership. Quantitative data on disease and health outcomes is available country-wide through District Health Information System 2 (DHIS2) and Demographic and Health Survey (DHS) databases (see Table 1) [26].

In Burkina Faso, primary data collection was conducted in three gold mining sites: (1) Houndé Mine in Houndé district (population in 2006: 77,000); (2) Yaramoko Gold Mine in Bagassi district (population in 2006: 33,000); and (3) Bissa Mine in Kongoussi district (population in 2006: 71,000). The three mines have been operational since 2017, 2016, and 2013, respectively. While the Houndé and Bissa mines are open-pit mines, operations in Bagassi are underground. Houndé is a small city located approximately 5 km from the mine with formal and informal settlements reaching closer. In Bagassi, villages are scattered around the mining premises. Sabcé is the closest town from the Bissa mine. During the field visit, artisanal mining sites were observed around all three mines.

In Ghana, primary data collection was conducted in three gold and manganese mining sites: (1) Edikan Gold Mine (Perseus Mining) in the Upper Denkyira West district (population in 2019: 71,425); (2) Tarkwa (Anglo Gold Ashanti, Gold Fields, and Ghana Manganese Company) in the Tarkwa-Nsuaem Municipal district (population in 2019: 117,550); and (3) Newmont Ahafo Mine (Newmont Goldcorp) in the Asutifi North Municipal (population in 2010: 52,259). The three mines have been operational since 2011, 1961, and 2003, respectively. All three are open-pit mines. Edikan Gold Mine has four nearby communities—Ayanfuri, Fobinso, Nkonya, and Abenabena—located in both the Upper Denkyira West and Amenfi West districts. The sites near Tarkwa include the small communities of Akoon, Tarkwabanso, Wangarakrom and Badukrom, and Iduaprim. Four communities are near the Newmont Ahafo Mine: Tutuka, Kenyase 1, Kenyase 2, and Ntrotroso.

In Mozambique, primary data collection was conducted in three types of mining sites—ruby, titanium, and coal—involving communities of four administrative districts and four mining companies: (1) Montepuez Ruby Mining (MRM) in Montepuez district (population in 2017: 261,235); (2) Kenmare Moma Mining in Larde district (population in 2017: 85,971) and Moma district (population in 2017: 310,706); (3) Vale Mozambique; and (4) International Coal Ventures (ICVL), the latter two in Moatize district (population in 2017: 343,546). All four are open-pit mines that have been operating since 2007 (Kenmare Moma Mining) and 2011 (MRM, Vale, and ICVL). MRM is surrounded by four small villages—N´sewe, N´thorro, M´pene, and Namanhumbir—whose main economic activities are agriculture and unregulated artisanal mining. Near Kenmare Moma Mining, Moma and Larde are the main and small emerging coastal towns located approximately 80 km and 20 km from the Kenmare mining company, respectively. The company activities affect communities from Moma district (ie, Pilivili locality) and Larde district (ie, Topuito locality). The eight affected villages at Pilivili locality (ie, Moma district) are the closest, located 5 km away from the mining company. There are 11 neighborhoods and villages affected by Kenmare Moma Mining, of which seven are located within the mining concession area and four are less than 100 meters from the mining pit: Tipane, Mutiticoma, Izoua, and Topuito. Agriculture and fishing are the main economic activities; however, commerce is an activity emerging mainly after mining implementation in 2005. Moatize is a small town 20 km from the city of Tete. It is an industrial complex composed of at least six large-scale coal mining companies—Vale Mozambique, ICVL, Nkodezi Coal Company, Minas de Revubue, Minas de Moatize Riversdale Mozambique Limitada, and Jindal Mozambique Minerals (JSPL)—some being subsidiaries of Vale Mozambique. At the time of site visit, very active commerce activities were observed along the main road. Apart from the town of Moatize, there are 12 small affected communities surrounding mining companies, four belonging to Moatize-sede locality—Catete, Mphandue, Matambanhama, and Ntchenga—and eight to Benga locality—Cancope, Capanga Gulo, Campanga lowane, Chitambo, Chitondo, Nyambalualu, Kangale, and Benga-sede. The last five communities are along the Zambeze River, the main local source of water. Agriculture and fishing are the main economic activities.

In Tanzania, three gold mining sites were chosen for fieldwork: Geita Gold Mine (Geita district), Buzwagi Gold Mine (Kahama district), and Bulyanhulu Gold Mine (Mslala district). Geita and Buzwagi are open-pit mines, while Bulyanhulu is an underground mine (based on observation). At the time point of data collection (ie, March to May 2019), the Geita Gold Mine was fully operational and the Buzwagi and Bulyanhulu mines were both in reduced production status, meaning that they were processing already extracted material and no longer extracting new raw material. The Geita Gold Mine, about 70 km south of Lake Victoria, is located between several villages and Geita, a main city of the district. The Buzwagi mine is surrounded by several villages and is 6 km away from Kahama, the capital of the district. The vibrant villages of Kakola, Bushing’we, and Kakola Namba 9 are next to the Bulyanhulu mine. During the field visit, artisanal mining sites were observed around all three mines.

**Table 1:** Primary quantitative outcomes and data availability from the District Health Information System 2 (DHIS2), the Demographic and Health Survey (DHS), and other national-level databases.

Primary quantitative outcomes		Available in the DHIS2 at the district level	Available at the district level through the DHS or another data source
**Health-related SDG^a^ target indicator**			
	Stunting rate among children below the age of 5 years	Burkina Faso Ghana Mozambique Tanzania	Burkina Faso Tanzania
	Maternal deaths per 100,000 live births	Burkina Faso Ghana Mozambique Tanzania	Burkina Faso Mozambique Tanzania
	Proportion of births attended by skilled health personnel	Burkina Faso Ghana Mozambique Tanzania	Burkina Faso Mozambique
	Under-5 mortality rate (deaths per 1000 live births)	Burkina Faso Ghana Mozambique	Burkina Faso Mozambique Tanzania
	Number of new HIV infections per 1000 uninfected population members (by age group, sex, and key populations)	Burkina Faso Ghana Mozambique Tanzania	Mozambique
	Tuberculosis incidence per 1000 persons per year	Burkina Faso Mozambique	Burkina Faso Mozambique Tanzania
	Malaria incident cases per 1000 persons per year	Burkina Faso Ghana Mozambique	Mozambique Tanzania
	Rates of noncommunicable diseases	Burkina Faso Ghana Mozambique	Mozambique Tanzania
	Number of road traffic fatal injury deaths within 30 days, per 100,000 population members (age-standardized)	Burkina Faso	Burkina Faso Ghana Mozambique Tanzania
	Health worker density and distribution	Burkina Faso	Burkina Faso Ghana Mozambique Tanzania
**Additional health indicators for monitoring health for the SDGs and health system performance**			
	Number and distribution of health facilities per 10,000 population members	Burkina Faso Mozambique	Burkina Faso Ghana Mozambique Tanzania
	Number of health workers per 10,000 population members	Burkina Faso	Burkina Faso Ghana Mozambique Tanzania
	Number of outpatient visits per 10,000 population members per year	Burkina Faso Mozambique	Burkina Faso Mozambique Tanzania
	Vaccination coverage in children aged 12-23 months	Burkina Faso Ghana Mozambique Tanzania	Burkina Faso Ghana Mozambique Tanzania
	Acute respiratory disease rate in children aged under 5 years	Burkina Faso Ghana Tanzania	Burkina Faso Ghana Mozambique Tanzania
	Diarrhea rate in children aged under 5 years	Burkina Faso Ghana Mozambique Tanzania	Burkina Faso Ghana Mozambique Tanzania
	Helminthic infection rate in different age groups	Burkina Faso Ghana	Burkina Faso Ghana Mozambique
	Syphilis rates in children and pregnant women	Burkina Faso Ghana Mozambique	Burkina Faso Ghana Mozambique
	Anemia rate in children aged under 5 years and pregnant women	Burkina Faso Ghana Mozambique Tanzania	Burkina Faso Ghana Mozambique Tanzania
	Hypertension rate in adults	Burkina Faso Ghana Tanzania	Burkina Faso Ghana Mozambique Tanzania
	Chronic respiratory tract infections rate among different age groups	Burkina Faso Tanzania	Burkina Faso Mozambique Tanzania

^a^SDG: Sustainable Development Goal.

### Quantitative Study Components

The main quantitative component of the study will be a retrospective observational longitudinal study using routine health data extracted from DHIS2 and other national-level databases. Specifically, this quantitative part of the study seeks to answer the following questions: (1) Which districts have been directly, indirectly, or not impacted by projects? (2) What differences in health-related SDG indicators and other health indicators (eg, health systems) can be observed in districts impacted by resource extraction projects compared to nonimpacted districts, including maternal and child health–related indicators? (3) What are the costs and benefits to the health system of project implementation? (4) How do projects impact on environmental determinants of health? and (5) What are the strengths and limitations of the national, routine, health information systems and other data sources and repositories at the national level to monitor how projects impact on health-related SDG target indicators and other health indicators? The specific research topics belonging to this work package are effects on health systems, morbidity and mortality, environmental determinants of health, maternal and child health, and health economics. The research in this work package uses existing DHIS2 surveillance data routinely collected by the governments of the study countries, along with supplementary national datasets—DHS, Service Availability and Readiness Assessment (SARA), Malaria Indicator Survey (MIS), Multi-Indicator Cluster Survey (MICS), AIDS Indicator Survey (AIS), and Service Provision Assessment (SPA) (see Table 2)—and remote sensing data (eg, geographical positioning, weather, and pollution data).

**Table 2:** Sources of existing national-level data used in the HIA4SD (Health impact assessment for sustainable development) study.

Data source	Abbreviation	Description	Data frequency
District Health Information System 2	DHIS2	The DHIS2 is a Web-based, open-source, health information management and visualization system used in more than 40 countries, including governmental agencies in the project countries. The DHIS2 databases contain information about many of the health-related Sustainable Development Goals (SDGs), as well as other key health indicators for monitoring health and health system performance.	Monthly
Demographic and Health Survey	DHS	The DHS is a nationally representative household survey that provides information on a wide range of health, population, and nutrition indicators.	Usually every 5 years
Service Availability and Readiness Assessment	SARA	The SARA is a health facility assessment survey designed to monitor service readiness and availability indicators.	Every 2-3 years (country dependent)
Malaria Indicator Survey	MIS	The MIS is a household survey designed to collect information on a wide range of malaria indicators.	Every 3-5 years (country dependent)
Multi-Indicator Cluster Survey	MICS	The MICS is a household survey designed to collect data on indicators related to the situation of children and women.	Every 5 years (country dependent)
AIDS Indicator Survey	AIS	The AIS is a household survey designed to collect information on indicators related to HIV/AIDS.	Every 5-6 years (country dependent)
Service Provision Assessment	SPA	The SPA is a health facility assessment survey designed to evaluate health service delivery.	Every 9-10 years (country dependent)

### Data Analysis

#### Geographic Information System Mapping Data

As part of the project, information on natural resource extraction projects (ie, type, size, and associated projects) and their exact geographic position will be extracted from government and other public record databases in the four study countries and mapped using ArcGIS software (Esri), a geographic information system (GIS). The data exist in diverse sources in different countries (ie, Ministries of Land and Natural Resources, Ministries of Water and Sanitation, Ministries of Science and Environment, Ministry of Energy, Forest Commissions, and local government records) as well as international monitoring organizations (ie, the International Finance Corporation [IFC] and the World Bank). Satellite imagery from the Landsat database will be used to estimate the impact of mining on settlement growth. The extracted satellite scenes will cover a time range spanning the years prior and after the opening of selected large-scale mining projects. With the aid of ancillary ground-truth information from historic Google Earth imagery, the size of settlements will be determined annually using machine learning algorithms. The annual growth of settlements will then be compared between mining and comparison sites.

#### Sampling of Natural Resource Extraction Project–Impacted Districts and Matching Comparison Districts

To analyze the differences in health outcomes between districts with and without extractive industries, a matched geographical analysis will be performed within the framework of the HIA4SD project to provide a stratified sampling framework. In a first stage, natural resource extraction projects will be mapped in each country together with their key attributes (ie, size and number of workers, length and age, type, and geographical footprint of the project). Each country will be spatially stratified into four levels for sampling purposes: (1) areas in direct proximity to a project (*highly impacted* areas), (2) areas within a 20-30 km buffer zone of the directly impacted regions (*impacted* areas), (3) regions bordering project areas that contain access roads or other economic or physical links to the project (*potentially impacted* areas), and (4) regions greater than 30 km away from a project that do not contain access roads or other economic or physical links to the project (*nonimpacted* areas). Comparison study sites will be selected from the *nonimpacted* areas and matched with important baseline characteristics (ie, community socioeconomic activities, vegetation, altitude, and ecological zone) to the *highly impacted* areas. All facilities, including public and private, that fit within perimeter boundaries and are registered in the DHIS2 database will be selected. In a second stage, *highly impacted* districts will be matched to two or three *nonimpacted* comparison districts within each country. Districts will be matched based on important baseline characteristics (ie, population, urbanization and aggregate night satellite brightness, square area, number of health care facilities, and disease rates) during the year before project implementation in the *highly impacted* district.

#### Quality Assessment of District Health Information System 2 Data and Association of Health Indicators With Natural Resource Extraction Projects

In order to assess the quality and completeness of the DHIS2 data, a comparison of DHIS2 and other data repositories with health statistics being collected at the local level under other work packages will be done. Next, potential positive and negative associations between health outcomes and health determinants (independent variables) and the existence of natural resource extraction projects (dependent variable) will be analyzed in the fourth working step by means of quasi-Poisson regression models and binomial regression models. The time span to be analyzed will be determined by the development history of the projects of interest in combination with the availability of data, which will vary between datasets. For the regression model, setting-specific cluster effects (eg, urban, rural, and type of project) will be taken into account. In order to maximize statistical power, pooled cross-country analysis and meta-analysis will be employed in addition to country-specific evaluations. Finally, based on the previous working steps, strengths and limitations of the national routine health information systems and other datasets at the national level to monitor impacts on health-related SDG target indicators and other health indicators of extraction projects will be determined and systematically reported [27].

#### Economic Cost-Benefit Analysis

Costs to the health system and to the households arising from prespecified disease conditions in each country will be estimated based on a retrospective approach. Health system costs will be collected from published information and on-site in selected health facilities in impacted districts through key informant interviews. Cost-generating components, such as required medical resources related to the corresponding illness, will be identified and a monetary value will be attributed to them. Costs incurred by the households, which are associated with the health care received, will be obtained through exit interviews in health facilities. The combination of excess cases associated with the presence of resource extraction projects (ie, data generated in the quantitative part of the study) and corresponding costs in each study country will allow the estimation of the cost incurred to the health system and households. A cost-of-illness analysis will be employed for estimating the costs incurred because of specific diseases or conditions (eg, HIV incidence rates, accidents, and chronic respiratory diseases) that have been identified in the first work package as being significantly increased due to the presence of extractive industry projects. The comparison of incidence data from *impacted districts* with incidence data from matching *comparison districts* (ie, districts with similar characteristics as *impacted districts* but without the presence of extractive industries) will allow for calculating the number of excess cases (eg, per 100,000 inhabitants) over the duration of 1 year. Probabilistic sensitivity analysis will be employed to allow for uncertainty around the cost estimates. Economic benefits of resource extraction projects will be measured monetarily in terms of direct and indirect financial contributions from the projects to the health sector. Financing of health infrastructure is considered to be a direct financial contribution, while the share of the health budget in the incremental tax revenues is considered to be an indirect contribution. We will also measure any other contributions.

#### Qualitative Data Analysis

The key informant interviews, focus group discussions, and in-depth interviews will be recorded using digital voice recorders for subsequent transcription. The transcripts will then be imported into software for qualitative data analysis—NVivo (QSR International)—to code the transcripts for thematic content and framework analysis based on the COREQ (COnsolidated criteria for REporting Qualitative research) criteria [28,29]. The information obtained from different sources will be used for systematically assessing the different topics of the PhD projects (ie, partnership arrangements and the perception of health care services availability and accessibility), with a particular angle on maternal and child health, sexual and reproductive health of adolescent girls, and key social determinants linked to the perceived health impacts.

#### Triangulation of Quantitative and Qualitative Results

Using triangulation methodologies, the most striking quantitative and qualitative results will be compared within and across the research topics and countries. Synergies and discrepancies in national data sources and local perspectives will be identified and used to develop new research questions and tools. In addition, the qualitative results will be used to explore whether or not the national-level data adequately capture the full range of health impacts from resource extraction projects as reported by the local community.

## Results

Fieldwork for the study began in February 2019 and concluded in February 2020. At the time of submission, qualitative data collection had been completed in Burkina Faso and Tanzania and is ongoing in Mozambique and Ghana. In Burkina Faso, 36 focus group discussions and 74 key informant interviews were conducted in three sites. In Ghana, 34 focus group discussions and 64 key informant interviews were conducted in three sites. In Mozambique, 75 focus group discussions and 103 key informant interviews were conducted in four sites. In Tanzania, 36 focus group discussions and 84 key informant interviews were conducted in three sites. Quantitative data extraction and collection is ongoing in all four study countries. Ethical approval for the study was received in all four study countries prior to beginning the fieldwork. Data analyses are underway and results are expected to be published in 2020 and 2021.

## Discussion

The interlinkages between health, environment, social equity, politics, and economy are extraordinarily complex in regions dominated by extractive industries. To investigate these intersectoral dependencies, we have designed an innovative new study that is both mixed methods and multi-focus, allowing for a flexible research design to address many specific research questions under the umbrella of HIA. By utilizing a mixed methods design that triangulates both quantitative and qualitative findings, the study offers the opportunity to look at the impacts of natural resource extraction projects on population health from many different approaches and vantage points, allowing for a more complete understanding of the social, institutional, and economic changes that follow implementation of a new extractive project. This type of research cannot exist in an academic vacuum; to ensure sustainable policy development we have also incorporated the engagement of key stakeholders in every phase of the study with the aid of research on national and international governance, with active participation at all levels of the host country from policy and political leaders at the national level to everyday community members affected by resource extraction projects. Long after the study has concluded, a new resource of HIA will remain in-country in the form of highly trained PhD candidates identified by the partner institutes that are already active in the health research of their countries.

The study has many strengths stemming from its novel design, in particular the complementary nature of the data collection due to the mixed methods design. By integrating many data sources and types, including national-level datasets, household data collected by international bodies, and primary data collected by diverse qualitative and quantitative methodologies, the study strengthens its evidence base while avoiding relying too much on one data source that may be biased or characterized by missing data. In particular, the in-depth multi-focus qualitative research across the four study countries means that the study captures a complete picture of health “on the ground” in diverse regions affected by natural resource extraction, instead of relying only on national-level data sources. The participatory and flexible approach to the study design means that the specific research topics and questions were largely driven by the ideas of researchers in the partner countries themselves, as opposed to a traditional top-down approach. This design feature ensures that findings will be relevant to policy makers at the national level, instead of mirroring priorities of the more distant international research community. Finally, instead of a siloed research approach where all researchers work separately to gather their own data and analyze it, data will be generated and shared across all research countries and topics, enabling within- and between-country comparisons. This multidisciplinary research will generate new hypotheses and findings across a wide range of health concerns, while promoting HIA methodology as an underutilized resource.

The DHIS2 database deserves special mention here as a new solution to data management across sub-Saharan Africa that is still relatively underutilized, despite being widely available for the past 5 years. By extracting and analyzing a wide range of health indicators countrywide for the past 5 years in all study countries, our study aims to realize the potential of DHIS2 in opening a new era of data being generated and analyzed in-country. This aim has particular reverberations for enabling countries using DHIS2 to start tracking their own progress toward the SDGs, instead of relying on estimates from international bodies.

There are some potential limitations to our study. The DHIS2 database is a relatively new data source, and little is known about the quality of its data. In addition, DHIS2 represents individuals that sought medical care from health care facilities and is likely underreporting actual incidence of disease, especially in areas without a strong reporting system or for less-severe disease that is not always treated in formal health care facilities. For some resource extraction projects, routine DHIS2 data may not be available before project implementation, making it difficult to approximate a true baseline health level in the impacted district. However, trends over time can still be described. In addition, nonimpacted districts that are matched as closely as possible on key demographic characteristics to impacted districts will provide a comparison that avoids the need for a baseline. These limitations are outweighed by the potential benefit of a comprehensive longitudinal assessment of health and extractive industries for the first time. In addition, as part of the study a quality assurance analysis will be done to evaluate the strengths and weaknesses of this data, which will aid researchers to better understand any biases or missing data that may affect the findings and also potentially identify areas for improvement in public health surveillance in the study countries. Finally, the primary quantitative data collection will be cross-sectional and, therefore, inferences about causality will be limited, although this will remain an important insight in an area where little previous research has been done. The qualitative research will be, by necessity, of a nonrandom sample of impacted community members, and the use of a “gatekeeper” for recruitment may result in a nonrepresentative sample; however, by recruiting across many communities and countries and using multiple gatekeepers where possible, the potential for bias will be limited as much as possible. By including a diversity of data sources in our study, many of these limitations will be reduced or eliminated.

The HIA4SD study is an innovative study designed to give a more complete picture, to our knowledge than ever before, of population health in regions affected by extractive industry activity. This will allow host countries to move closer to sustainable development by harnessing the potential positive socioeconomic impacts of these projects while minimizing negative consequences due to migration, disease, and environmental changes. By taking a thematic approach that incorporates a multifaceted mixed methods research design, using both already-existing secondary data to track change in project-impacted regions over time and primary data collected in the field, the project is able to capture the perspective of the many actors that have a stake in sustainable development in these regions while reducing project reliance on any one data source that may be incomplete. Finally, the integration of research on governance systems and engagement with key stakeholders into the project from the beginning helps situate and translate our research findings into concrete policy recommendations, aiding progress toward the SDGs.

## Abbreviations

AIS: AIDS Indicator Survey
COREQ: COnsolidated criteria for REporting Qualitative research
DHIS2: District Health Information System 2
DHS: Demographic and Health Survey
GIS: geographic information system
HIA: health impact assessment
HIA4SD: Health impact assessment for sustainable development
ICVL: International Coal Ventures
IFC: International Finance Corporation
JSPL: Jindal Mozambique Minerals
MICS: Multi-Indicator Cluster Survey
MIS: Malaria Indicator Survey
MRM: Montepuez Ruby Mining
r4d programme: Swiss Programme for Research on Global Issues for Development
SARA: Service Availability and Readiness Assessment
SDC: Swiss Agency for Development and Cooperation
SDG: Sustainable Development Goal
SNSF: Swiss National Science Foundation
SPA: Service Provision Assessment
